# From squid giant axon to automated patch-clamp: electrophysiology in venom and antivenom research

**DOI:** 10.3389/fphar.2023.1249336

**Published:** 2023-08-24

**Authors:** Shirin Ahmadi, Melisa Benard-Valle, Kim Boddum, Fernanda C. Cardoso, Glenn F. King, Andreas Hougaard Laustsen, Anne Ljungars

**Affiliations:** ^1^ Department of Biotechnology and Biomedicine, Technical University of Denmark, Kongens Lyngby, Denmark; ^2^ Sophion Bioscience, Ballerup, Denmark; ^3^ Institute for Molecular Bioscience, University of Queensland, St Lucia, QLD, Australia; ^4^ Australian Research Council Centre of Excellence for Innovations in Protein and Peptide Science, University of Queensland, St Lucia, QLD, Australia

**Keywords:** electrophysiology, patch-clamp, ion channel, venom, antivenom, drug discovery, neurotoxin

## Abstract

Ion channels play a crucial role in diverse physiological processes, including neurotransmission and muscle contraction. Venomous creatures exploit the vital function of ion channels by producing toxins in their venoms that specifically target these ion channels to facilitate prey capture upon a bite or a sting. Envenoming can therefore lead to ion channel dysregulation, which for humans can result in severe medical complications that often necessitate interventions such as antivenom administration. Conversely, the discovery of highly potent and selective venom toxins with the capability of distinguishing between different isoforms and subtypes of ion channels has led to the development of beneficial therapeutics that are now in the clinic. This review encompasses the historical evolution of electrophysiology methodologies, highlighting their contributions to venom and antivenom research, including venom-based drug discovery and evaluation of antivenom efficacy. By discussing the applications and advancements in patch-clamp techniques, this review underscores the profound impact of electrophysiology in unravelling the intricate interplay between ion channels and venom toxins, ultimately leading to the development of drugs for envenoming and ion channel-related pathologies.

## 1 Introduction

Ion channels are specialized proteins that span the membrane, forming a pore or channel that allows charged ions to move across the membrane. They play a critical role in a wide range of physiological processes, including, but not limited to, neurotransmission and muscle contraction (Subramanyam and Colecraft, 2015). Activation of ion channels occurs through different mechanisms, such as in response to changes in membrane potential (voltage-gated channels) or due to binding of a ligand (ligand-gated channels). Voltage-gated ion channels come in various types, such as voltage-gated sodium (Na_V_) and voltage-gated potassium (K_V_) channels, and they are responsible for multiple physiological functions. For instance, in excitable cells, Na_V_ channels play a crucial role in the initial depolarization of membrane and the generation of action potential, while K_V_ channels contribute to membrane repolarization (Grider et al., 2023). Ligand-gated ion channels, on the other hand, are activated by specific ligands such as neurotransmitters. Acetylcholine, for instance, is an excitatory neurotransmitter found throughout the nervous system, which upon binding to nicotinic acetylcholine receptors (nAChRs) causes sodium and potassium ions to flow across the membrane, resulting in membrane depolarization and triggering of an action potential (Zoli et al., 2018).

Due to the multiple vital functions of most ion channels, molecules targeting these channels can interfere with and impair fundamental physiological mechanisms. This is something that venomous creatures take advantage of by producing toxins that have evolved specifically to target ion channels, aiding in prey capture and protection against predators (Osipov and Utkin, 2017). A vast array of venom toxins has been identified to interact with both voltage-gated and ligand-gated ion channels. Many of the toxins that target voltage-gated ion channels have been extracted from arthropod venoms, such as those found in scorpions and spiders (Gilchrist et al., 2014; Diochot, 2021). In terms of venom toxins affecting ligand-gated ion channels (Nasiripourdori et al., 2011), the most extensively studied toxins are those derived from cone snail venoms (Arias and Blanton, 2000) and snake venoms (Barber et al., 2013). For example, a prominent category of ion channel-targeting toxins in snake venoms are α-neurotoxins (αNTxs) that target nAChRs in the nervous system, hindering neurotransmission and muscle contraction. Thereby, snake αNTxs may cause a wide range of pathophysiological effects in humans, from itching and sweating to paralysis and respiratory arrest (Nirthanan et al., 2016).

Given the significant medical complications caused by many ion channel-targeting toxins, as well as their potential for use as human therapeutics (King, 2011), understanding their mechanism of action is critical. However, the complex structure and function of both (neuro)toxins and ion channels necessitates complex experimental setups. In this regard, a powerful tool to study toxin-ion channel interactions in real-time is electrophysiology, particularly patch-clamp methodologies. Patch-clamp electrophysiology enables direct measurement of changes in ion channel activity upon toxin exposure, and it has played a fundamental role in unraveling the mode of action of ion channel-modulating toxins, as well as in determination of their potency and selectivity. Moreover, it has recently been reported that patch-clamp electrophysiology can be used to evaluate the neutralization capacity of antibodies against scorpion (Restano-Cassulini et al., 2017) and snake venoms (Lynagh et al., 2020; Ledsgaard et al., 2022; 2023; Miersch et al., 2022), facilitating the development of efficient therapies against envenomings caused by these animals.

Although patch-clamp electrophysiology is widely recognized and utilized in the field of toxinology and venom research, there appears to be a lack of comprehensive reviews focusing on its application in antivenom research. This gap may be attributed to the relatively recent integration of patch-clamp techniques in antivenom studies, resulting in limited literature on the subject. In this review, we first present the history of electrophysiology and patch-clamp methodologies in ion channel research. We then describe the fundamental role these techniques have played in venom and antivenom research, including their role in venom-based drug discovery pipelines and assessment of the neutralizing effect of antivenoms. Finally, we discuss how patch-clamp electrophysiology can be employed in the development of new types of therapies against snakebite envenoming.

## 2 History of patch-clamp and ion channel discovery

In 1945, Hodgkin and Huxley were the first to demonstrate the existence of voltage-gated ion channels in cell membranes and to discover the principles of ion channel function (Hodgkin and Huxley, 1945; 1952). They used the giant axon of the squid *Loligo forbesii* as a model system to study the electrical activity of nerve cells. They found that nerve impulses are initiated by the movement of ions across the cell membrane, mediated by conducting structures that we now know as ion channels. Hodgkin and Huxley introduced mathematical equations that are still used for electrical membrane modelling and showed that the gating (opening and closing) of ion channels is controlled by changes in membrane potential, and that the resulting ion fluxes generate the electrical signals that underlie nerve impulses. For this discovery, they were awarded the Nobel Prize in Physiology or Medicine in 1963. Furthermore, together with Cole (Cole and Curtis, 1939), Hodgkin and Huxley developed the voltage-clamp technique, which was pivotal for the development of patch-clamp.

**FIGURE 1:**
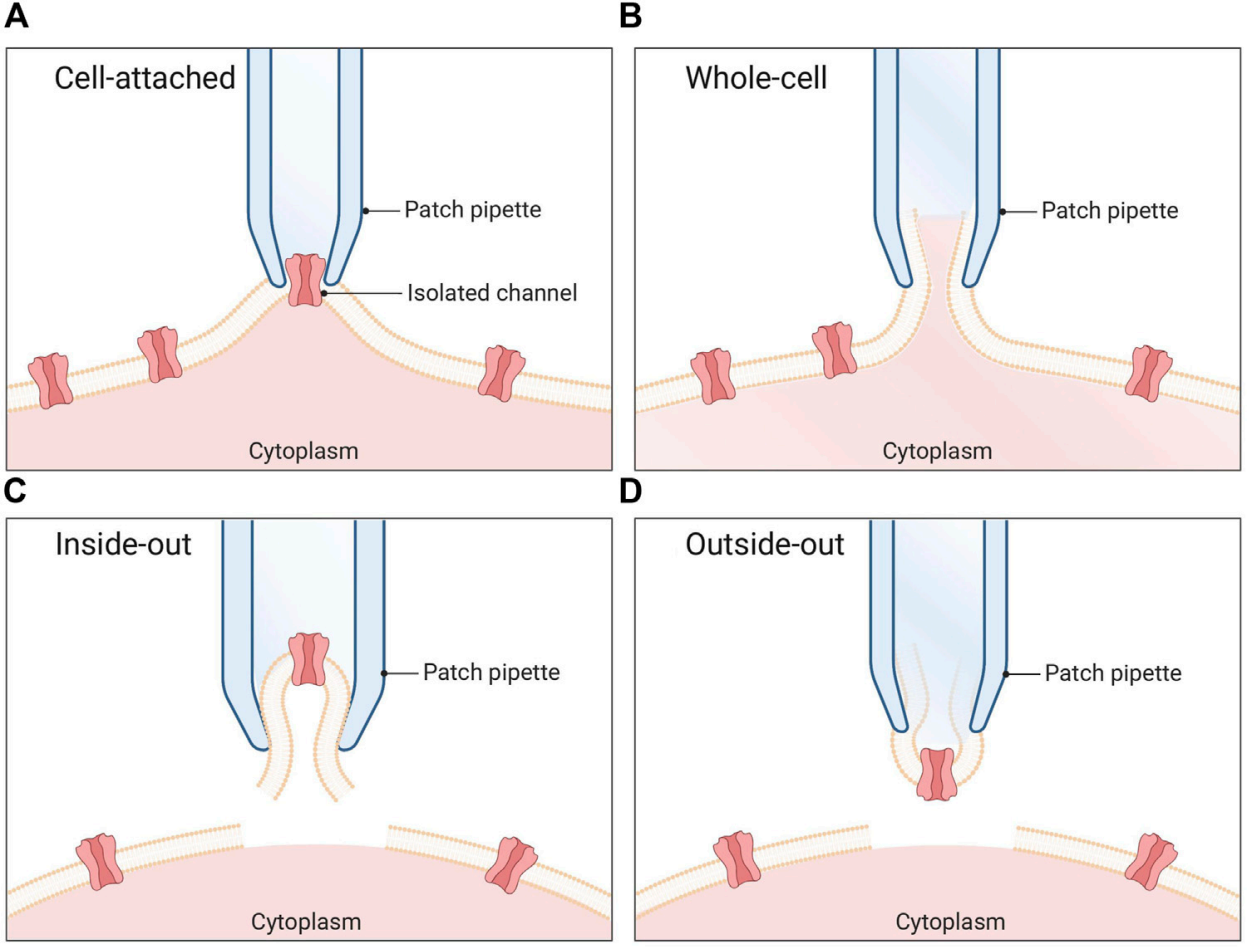
Patch clamp configurations. **(A)** Cell-attached mode: The pipette is positioned in proximity to the cell membrane, and gentle suction is applied to establish a tight seal between the pipette and the membrane. This configuration enables the recording of single channel currents. **(B)** Whole-cell mode: A pipette is utilized to apply a sufficiently strong suction force, resulting in the rupture of the cell membrane. Consequently, the pipette gains access to the cytoplasmic environment of the cell. **(C)** Inside-out mode: The membrane patch is carefully excised from the cell and immersed in the surrounding bath solution. In this arrangement, the seal remains intact while the rest of the cell is disrupted. **(D)** Outside-out mode: Initially, the whole-cell method is employed, followed by the retraction of the pipette after membrane rupture. This process causes a section of the membrane to detach from the cell and reconfigure into a small, vesicular structure, with the external side of the membrane exposed to the bath solution.

In the 1970s, Neher and Sakmann built on the work of Hodgkin and Huxley to introduce the patch-clamp technique (Neher et al., 1978). Using a glass pipette with an opening of a few micrometers, they were able to obtain direct measurements of ion channel currents across the cell membrane (Erwin Neher and Sakmann, 1976). By gently poking a micropipette into a cell membrane and applying a low, negative pipette pressure, they could create a high electrical resistance seal, named a giga seal as the resistance is measured in giga-ohms. Based on this cell-attached conformation (Figure 1A), further manipulation of the membrane give rise to whole-cell (Figure 1B), inside-out (Figure 1C), and outside-out (Figure 1D) configurations. In a whole-cell configuration, Neher and Sakmann applied a rapid, high-pressure suction to the pipette to rupture the membrane and create electrical contact with the intracellular environment. This allowed them to measure the electrical activity of the cell with great accuracy, manipulate the electrical activity of the cell, and control the voltage (Neher and Sakmann, 1976). The patch-clamp technique revolutionized the field of electrophysiology and won Neher and Sakmann the Nobel Prize in Physiology or Medicine in 1991.

**FIGURE 2:**
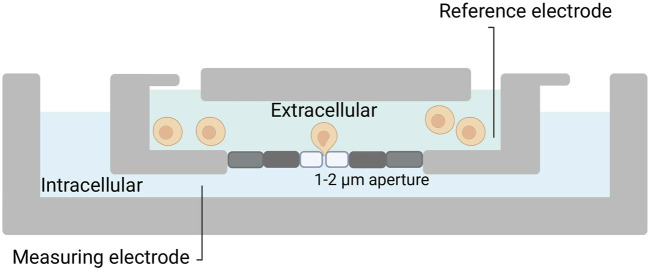
Planar patch-clamp. Silicon or plastic-based micro-fabricated substrates are utilized. Cells in suspension are added to the chip, and with a negative pressure, a cell is placed on top of the patch hole. Thereafter, the process is similar to manual patch-clamp.

While the patch-clamp techniques were groundbreaking, they were also time-consuming and required highly skilled operators, as each cell must be individually patched with a micropipette. To increase throughput and reproducibility, automated versions of the patch-clamp technique were needed (Wang and Li, 2003). Attempts to automate the process started in the late 1990s in Denmark, where the patch clamp setup was equipped with computer-controlled patch pipettes in combination with a visual recognition system and a series of rotating patch chambers (Asmild et al., 2003; Grass et al., 2003). Pipette-based automated patch-clamp techniques continue to exist and offer the potential for streamlining the otherwise time-consuming and limited-throughput process of patch-clamp electrophysiology within tissue slices. For instance, a group at Georgia Institute of Technology has developed an automated patch clamp platform, which can patch in brain slices with intact neuronal networks (Kolb et al., 2019). Nevertheless, automated patch-clamp (APC) picked up speed in the early 2000s, with the introduction of chip-based systems, which enabled researchers to perform planar patch-clamp (Wang and Li, 2003). Here, the microchips have laser-drilled holes (1-2 µm) in a planar substrate instead of a pipette; this creates giga seals that allow recording of picoamperes of currents from a single cell (Figure 2). This technique allows to run hundreds of individual patch-clamp experiments in parallel (Brüggemann et al., 2003; Kiss et al., 2003).

The development of automated approaches has significantly increased the throughput of the patch-clamp electrophysiology, as these systems are not only automated, but can also simultaneously record measurements from multiple cells, which allows for faster and more efficient data collection. The first APC systems introduced in the late 1990s were used for drug discovery in the pharmaceutical industry. These early systems were able to measure currents from four to eight cells simultaneously, with a throughput of approximately 100–200 cells per day (Dale et al., 2007). Since then, the throughput of APC has continued to increase with advances in technology. In the early 2000s, APC systems were able to record from up to 48 cells simultaneously (Willumsen et al., 2003), and today, this number has increased to 384/768 cells (Obergrussberger et al., 2015; Chambers et al., 2016), enabling a throughput of thousands of experiments per day (Li et al., 2022). Various APC platforms are commercially available, however, a comprehensive review of such platforms falls beyond the scope of this manuscript and can be found elsewhere (Bell and Dallas, 2018).

The increased throughput of APCs has made them a valuable tool not only for drug discovery and high-throughput screening in the pharmaceutical industry, but also for basic research across several disciplines, from neurological (Rosholm et al., 2022; Ghovanloo et al., 2023) and cardiovascular sciences (Bell and Dallas, 2021) to venom (Olamendi-Portugal et al., 2008) and antivenom research (Restano-Cassulini et al., 2017; Ledsgaard et al., 2022; 2023; Miersch et al., 2022).

## 3 Electrophysiology in venom research

Electrophysiology techniques have played a significant role in advancing venom research, especially for elucidating the mechanisms by which venom toxins interact with and modulate ion channels and for the identification of toxins that can be used as scaffolds for drug development. The first, and perhaps the most important, of these techniques is the two-electrode voltage clamp (TEVC) electrophysiology method, used by Hodgkin and Huxley in their study of the squid giant axon. In this technique, two intracellular electrodes (one voltage sensor and one current injector) are used to set the cell membrane potential to a desired value and to record the current across the membrane. By applying this method to oocytes from the African clawed frog, *Xenopus laevis*, various toxins that act on a wide range of ion channels, from the Na_V_ and K_V_ channels to ligand-gated nicotinic acetylcholine (nACh) and gamma aminobutyric acid (GABA) receptors (Collins et al., 2009; Kvist et al., 2011; Bertaud et al., 2022), have been studied. The results of these studies provided valuable insights into how some toxins affect ion channel conductance, activation/inactivation dynamics, and opening/closing thresholds. Additionally, because of the low abundance of endogenous ion channels within their cell membrane, *X. laevis* oocytes serve as a versatile model for the expression and functional characterization of exogenous ion channels (Lin-Moshier and Marchant, 2013). Beyond expression of wild type ion channels, the oocytes can be used for expression of ion channels with modified subunit compositions or mutated variants, thereby serving as a powerful tool to investigate the interaction between venom toxins and ion channels. For instance, Ellinor *et al.* used this system to describe the structural determinants of the blockade of N-type voltage-gated calcium (Ca_V_) channels (*i.e.,* Ca_V_2.2) by ω-conotoxin-GVIA, originally described in the venom of *Conus geographus* (Olivera et al., 1984). Here, the researchers created chimeric channels*,* containing individual motifs from both a Ca_V_2.2 channel and a ω-conotoxin-GVIA insensitive channel, and expressed these in *Xenopus oocytes*, which allowed them to identify the binding sites for the toxin on the ion channel (Ellinor et al., 1994). Information from such studies is important, as the identification of ion channel binding sites for a toxin can help provide an explanation for the remarkable capability of many toxins to distinguish between different isoforms and subtypes of ion channels (Chassagnon et al., 2017). In turn, discovery of toxins with high ion channel specificity can serve as a starting point for drug development efforts, possibly by using the toxin as scaffold or benchmark molecule. This approach has led to the discovery of ziconotide (Prialt^®^), a drug chemically identical to the ω-conotoxin-MVIIA peptide from *Conus magus* (Miljanich, 2004; Safavi-Hemami et al., 2019). By applying whole cell patch-clamp electrophysiology, it was demonstrated that ω-conotoxin-MVIIA, in contrast to ω-conotoxin-GVIA, can distinguish between Ca_V_2.1 and Ca_V_2.2 subtypes of N-type Ca^2+^ channels (Safavi-Hemami et al., 2019). This specificity is important since, in mammals, Ca_V_2.1 is expressed at the neuromuscular junction, whereas Ca_V_2.2 is exclusively expressed at the dorsal horn of the spinal cord. In this context, Ca_V_2.2 plays an essential role in the release of neurotransmitters at the presynaptic termini of pain fibers that connect with spinal cord neurons. Consequently, when the ω-conotoxin-MVIIA peptide binds to Ca_V_2.2, it effectively prevents the transmission of signals from the pain fibers to the central nervous system, while exerting minimal or no effect on neuromuscular junctions (Safavi-Hemami et al., 2019). Ziconotide has received approval for the treatment of chronic and cancer-related pain, as an alternative to, or in combination with, morphine (Staats et al., 2004; McGivern, 2007; Williams et al., 2008), showcasing the practical application of toxin-ion channel knowledge for therapeutic advancements.

Even though TEVC electrophysiology has played a major role in advancing our understanding of toxin-ion channel interactions, it is both time-consuming, technically challenging, and requires highly specialized operators. Therefore, automated versions of this technique have been developed that facilitate high-throughput studies of toxin-ion channel interactions. As an example from 2022, Nys *et al.* used automated electrophysiology, in combination with crystallography and ion channel mutagenesis, to determine the precise location of the interactions between a consensus elapid short-chain α-neurotoxin (ScNtx) and both the muscle subtype and the α7 neuronal subtype of nAChR (Nys et al., 2022). While the ScNtx showed a similar inhibitory effect on both nAChR subtypes, the recovery process after inhibition was significantly different for the two receptor subtypes. The muscle subtype exhibited a slow and partial recovery, whereas the α7 neuronal subtype displayed an immediate and complete recovery, suggesting that the ScNtx has a higher binding affinity to the muscle subtype of nAChRs compared to the α7 neuronal subtype. In addition, the combination of mutagenesis data and electrophysiology experiments yielded compelling evidence showing that point mutations at the toxin-ion channel interface had a more pronounced effect on the α7 subtype than the muscle subtype (Nys et al., 2022). Such detailed knowledge on the mechanisms of action for different toxins is central for the development of effective antivenoms, as it aids the rational assessment of whether the function of a toxin can be blocked by different types of inhibitors.

Despite its wide utility, the use of TEVC electrophysiology is limited to the measurement of macroscopic currents across the membrane of large cells, which can pose limitations to recording fast-kinetics currents, such as sodium currents (Ivorra et al., 2022). In the 1970s, patch-clamp methodologies were developed, in which the substitution of the impaling electrodes of the two-electrode voltage clamp technique with a low electrical noise borosilicate glass electrode (patch pipette) in tight contact with the cell membrane allows for the measurement of signals in the pico ampere range and makes it possible to detect currents across the membrane of small cells or even single ion channels (Petkov, 2009). Among the multiple patch-clamp configurations, including cell-attached, whole-cell, inside-out, and outside-out modes, the whole-cell voltage clamp technique is the most widely used in venom research (Figure 1).

Whole-cell voltage clamp enables the analysis of microscopic currents through ion channels in their native environment in a variety of cell types. Moreover, transfection of mammalian cell lines, such as HEK or CHO cells (Cardoso et al., 2015), with ion channel encoding mRNA or DNA enables the expression of exogenous ion channels at significantly higher levels compared to native cells (Gamper et al., 2005; Ponce et al., 2018). For instance, Cardoso *et al.* performed an automated whole cell patch-clamp electrophysiology experiment, using a CHO cell line expressing the Na_V_1.7 isoform of the human voltage-gated sodium channel. This channel isoform plays a crucial role in pain signaling in humans and is considered a prominent target for therapeutic interventions in pain management. In their experiment, Cardoso *et al.* used high-throughput screening to identify potential Na_V_1.7-specific inhibitors present in the venoms from 40 species of tarantula spiders (*Theraphosidae* family), which resulted in the discovery of a 33-residue peptide from the venom of *Thrixopelma pruriens,* named µ-TRTX-Tp1a (ProTx-III). The results obtained from this whole-cell patch-clamp experiment indicated that this peptide preferentially inhibits the Na_V_1.7 without causing significant alterations in the voltage dependence of activation or steady-state inactivation. Thereby, this toxin distinguishes itself from the majority of spider toxins that typically modulate Na_V_ channels by prominently affecting the voltage dependence of activation or inactivation (Cardoso et al., 2015). The discovery of subtype-selective peptides like µ-TRTX-Tp1a is central for the identification of scaffolds that may be used as leads in drug development programs, as they are less likely to modulate other ion channel subtypes and thereby cause off-target effects.

The use of medium-throughput and high-throughput patch-clamp methods has been essential for the characterization of venom-derived drug candidates for treatment of diseases, such as epilepsy (Cardoso, 2020), irritable bowel syndrome (Cardoso et al., 2021), and endometriosis (Castro et al., 2023). Similar to the application of Na_V_ channel-expressing cell lines for the identification of molecules that could be used for pain management, other cell lines, such as the rhabdomyosarcoma cell line, which stably expresses the muscle subtype of nAChR, have successfully been employed for patch-clamp electrophysiology in antivenom research.

## 4 Electrophysiology in antivenom research

Although electrophysiology techniques were swiftly integrated into venom research following their invention, it took considerably longer before they were applied in the field of antivenom research. In fact, the application of electrophysiology techniques in the antivenom field did not start until 1994, when Harvey *et al.* suggested that a chick *biventer cervicis* nerve–muscle preparation could be used for evaluating the ability of antivenoms to neutralize the neurotoxic and direct myotoxic effects of venoms (Harvey et al., 1994). In the same year, Barfaraz and Harvey successfully utilized the chick *biventer cervicis* nerve–muscle preparation to compare the relative potencies of six different antivenoms for neutralization of neurotoxic and myotoxic proteins present in six distinct snake venoms (Barfaraz and Harvey, 1994). The chick *biventer cervicis* nerve–muscle preparations, along with mouse phrenic nerve-diaphragm preparations, have since then been employed to evaluate the efficacy of antivenoms in neutralizing the neuromuscular blocking effect induced by various venoms (Fry et al., 2001; Camargo et al., 2011; Kornhauser et al., 2013; Herrera et al., 2016; Silva et al., 2017; Madhushani et al., 2020; Zukifli et al., 2022).

While assays involving nerve-muscle preparations have proven to be highly valuable and effective, their implementation and integration into routine quality control analysis of antivenoms is hindered by their time-consuming and labor intensive nature (Gutiérrez et al., 2021). Therefore, other electrophysiological approaches, such as different patch-clamp methodologies, may be more attractive for researchers, as they offer more adaptability and versatility for evaluating the efficacy of antivenoms. As an example, in 2017, Restano-Cassulini *et al.* demonstrated that whole cell patch-clamp using cell lines with heterologous expression of human Na_V_ and nAchR could be used for validation of the neutralizing efficacy of scorpion antivenoms (Restano-Cassulini et al., 2017). Approaches like this hold the potential to be employed for the quality control of scorpion antivenoms and might provide an ethically preferable alternative over animal experiments, as they conform with the 3Rs principle of replacement, reduction, and refinement of animal experiments, encouraged by the World Health Organization (Gutiérrez et al., 2021). Another advantage of patch-clamp approaches is that cell lines that express human ion channels, either endogenously or via heterologous expression, can be used. Thus, data obtained from these approaches may prove to be clinically even more relevant than animal models, since there can be slight, yet potentially significant, differences between human ion channels and those found in animal cells. While it is necessary to further substantiate the results from the *in vitro* patch-clamp protocol suggested by Restano-Cassulini *et al.* and benchmark these quantitatively with the existing *in vivo* models, the *in vitro* patch-clamp technique could potentially be implemented as a high-throughput, standard approach for quality control of scorpion antivenoms and may find utility for the evaluation of other types of antivenoms as well.

Patch-clamp techniques not only facilitate the evaluation of the neutralization capacity of existing antivenoms, but can also aid in the development of new and improved types of antivenoms by enabling the functional characterization of recombinant and/or consensus toxins. One example of such an application is related to one of the most toxic super-families of snake venom toxins for mammals, *i.e.*, the three finger-toxin family. A subgroup of 3FTxs is the α-neurotoxins, which bind to nAChRs. Thereby, these toxins cause post-synaptic neurotoxicity through non-depolarizing blockade of the nAChRs and impair neuromuscular transmission. Due to their small size (6–8 kDa), low immunogenicity (Laustsen et al., 2017), and relatively low abundance in most venoms in which they occur, α-neurotoxins are often unable to provoke a strong immune response. As a result, they may not effectively be neutralized by current snake antivenoms, as these antivenoms are produced through hyper-immunization of animals (Laustsen et al., 2017). One approach for improving antivenoms against α-neurotoxins is thus by using designed consensus toxins with selected amino acid substitutions to enhance immunogenicity, which elicit an antibody response with broader cross-reactivity against multiple (related) toxins. As an example of this approach, de la Rosa *et al.* designed, expressed, and purified a consensus toxin, referred to as ScNtx, based on sequences from twelve short-chain α-neurotoxins found in the venoms of medically important snake species (de la Rosa et al., 2018). The pharmacological properties of ScNtx were thereafter assessed by voltage clamping, using *X. laevis* oocytes, which demonstrated the ability of ScNtx to antagonize the nAChR receptor. ScNTx was then used as an immunogen for the immunization of rabbits, whereupon their sera could recognize native short-chain α-neurotoxins present in various elapid venoms in an *in vitro* binding assay. This example demonstrates how patch-clamp can be used to establish the functionality of a recombinant (consensus) toxin and further facilitate the development of efficacious antivenoms derived through animal immunization.

Another approach that is being pursued to improve therapy against α-neurotoxins is the development of recombinant antivenoms (Kini et al., 2018). Here, monoclonal antibodies or nanobodies are typically discovered via *in vitro* display technologies using purified toxins (Ledsgaard et al., 2022; 2023; Miersch et al., 2022). However, the purification of low-abundance snake toxins, such as α-neurotoxins, from crude venoms is a challenging process involving intensive manual labor. Fortunately, it has recently been shown that this toxin purification process can be circumvented by using recombinant DNA technology. In 2023, Rimbault *et al.* cloned, expressed, and purified recombinant α-cobratoxin, a potent long-chain α-neurotoxins from *Naja kaouthia* venom (Rimbault et al., 2023), and demonstrated its ability to inhibit the ion flux through the muscular nAChR using a high-throughput automated electrophysiology platform, thereby confirming its correct folding. In brief, the human-derived rhabdomyosarcoma RD cell line with endogenous expression of αβγδ nAChR was used, and the current mediated by nAChR was elicited by the addition of acetylcholine. Afterwards, both recombinant and native α-cobratoxin were applied to the system, and it was shown that both could inhibit the acetylcholine-induced current in the cells. After confirming the functionality of the recombinant α-cobratoxin, the researchers used it as antigen in a phage display-based antibody discovery campaign and discovered three antibody fragments capable of blocking the ability of the native toxin to bind to the nAChR.

**FIGURE 3:**
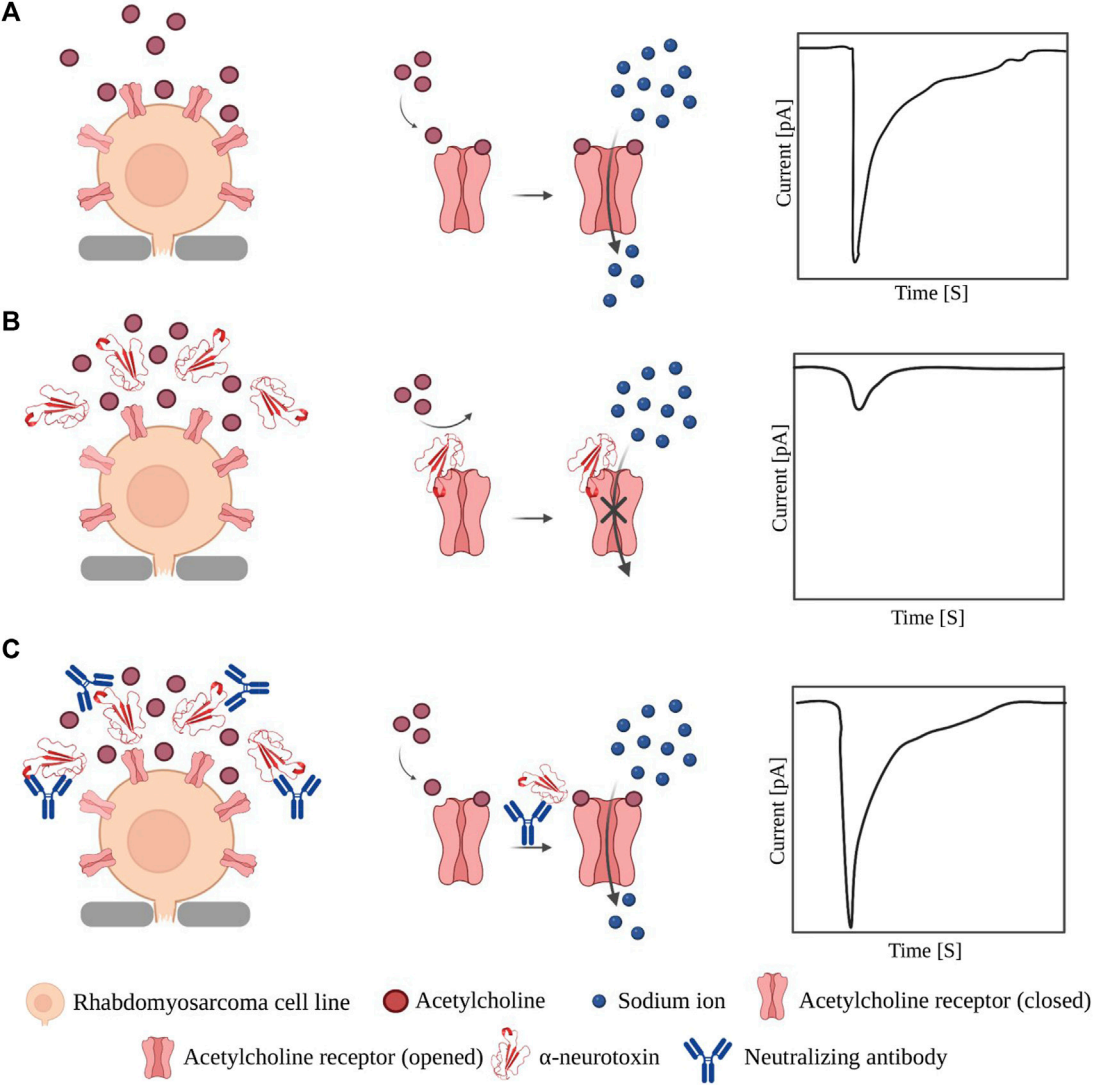
Application of patch-clamp in evaluating neutralization effect of binders against α-NTx. **(A)** Opening of nAChR upon binding of acetylcholine allows influx of sodium ions which can be measured as a current by patch-clamp instruments. **(B)** When α-NTxs are added to the cells, the pore and the influx of sodium ions are blocked, which results in a small current difference between the inside and outside of the cell. **(C)** When a mixture of pre-incubated α-NTx and a neutralizing molecule is added, α-NTxs cannot bind to the receptor, and the influx of sodium ions is not affected. Note: the relative sizes of the components in the figure are not accurately depicted.

Although the use of recombinant toxins offers several benefits, the technical challenges associated with their expression and purification (Rivera-de-Torre et al., 2022) hinder their widespread adoption. Therefore, crude venoms remain the primary sources of toxins used in antivenom research. Nevertheless, patch-clamp techniques still play an important role, particularly in the analysis of the neutralizing capacities of recombinant antibodies and other inhibitory agents discovered against ion channel-targeting toxins. For example, whole-cell patch-clamp techniques have been used to evaluate the functional inhibitory effect of synthetic antibodies (Miersch et al., 2022), chain-shuffled antibodies with native domains (Ledsgaard et al., 2022; 2023), and peptides (Lynagh et al., 2020) selected against native α-cobratoxin using phage display technology. In all these studies, the same principle was used for assessing the neutralizing potency, *i.e.,* the cell membrane current in a nAChR-expressing cell line was measured in three different scenarios: first, in the presence of acetylcholine alone (Figure 3A); second, after the addition of acetylcholine and toxin (Figure 3B); and finally, after the addition of toxin pre-incubated with the selected antibodies or peptides (Figure 3C).

In summary, these studies demonstrate that patch-clamp techniques not only facilitate the evaluation of the neutralization capacity of existing antivenoms, but also enable functional characterization of the native and recombinant toxins used for antivenom development, as well as high-throughput functional screening of monoclonal antibodies and other toxin-neutralizing agents.

## 5 Discussion

The application of electrophysiology, particularly APCs, for the study of venom toxins has significantly contributed to our understanding of ion channel pharmacology and function. Additionally, the utilization of diverse electrophysiology techniques to study toxin-ion channel interactions and screen venoms for specific ion channel-targeting toxins has played a crucial role in the discovery of venom-based therapeutic lead molecules for ion channel-related diseases, such as chronic pain, epilepsy, and irritable bowel syndrome. Moreover, within the field of envenoming therapy, electrophysiology techniques, such as patch-clamp, are now playing a pivotal role in advancing the development of novel types of antivenoms that can neutralize ion channel-targeting toxins.

It is, however, important to highlight that although APC systems have significantly advanced venom and antivenom research, they have not entirely replaced the need for manual patch clamp techniques. Rather, these two methods should be considered complementary. Manual patch clamp remains essential for successfully patching cells in some cell cultures and tissue preparations, such as organoids and brain slides with intact neural networks. On the other hand, planar automated patch clamp is well suited for cells in suspension, where the cell selection is unbiased, and operators cannot visually examine the cells as in manual patch clamp. Additionally, substantial cell line and assay optimization is often required before conducting high-throughput screens on APCs. In cases where the project’s throughput requirements do not justify the initial investment for an APC system, manual patch clamp offers a more accessible starting point (Dunlop et al., 2008; Bell and Dallas, 2018). In the field of venom research, a common limitation of employing manual patch clamp techniques arises from the scarcity and costliness of toxins. Meeting the substantial quantities required for perfusion systems used in manual patch clamp setups can be problematic. Conversely, numerous APC systems utilize microfluidics, necessitating a smaller volume and thereby low amount of materials per experiment. However, this reduction in liquid volume leads to a significant increase in the liquid-to-surface ratio, potentially giving rise to a challenge through nonspecific polyreactivity, “stickiness,” of some toxins (Bridgland-Taylor et al., 2006; Gillie et al., 2013; Yu et al., 2016).

In general, a limitation of electrophysiology techniques arises from their prerequisite for an intact cell membrane. This poses challenges to their application in venom and antivenom research, especially when studying animal venoms that contain membrane-disrupting components, such as cytotoxins and phospholipases. Consequently, certain patch-clamp configurations may not be immediately suitable for studying complex whole venoms, such as those found in most snakes, but require that laborious toxin expression and/or purification protocols are used to produce or isolate the ion channel-targeting components from the whole venom. In contrast, venoms with no or a low abundance of membrane-disrupting toxins, like scorpion venoms that mainly contain ion channel-targeting toxins, are more amenable to patch-clamp. This makes patch-clamp a valuable tool for evaluating the efficacy of scorpion antivenoms and may position patch-clamp as a potential alternative to animal experiments for quality control of antivenoms. The substitution of animal models with *in vitro* methodologies remains a topic of debate. It is challenging to fully assess how results obtained from *in vitro* patch-clamp experiments will translate to the clinical setting in terms of treatment outcomes. Therefore, it is crucial to comprehend the advantages and limitations of electrophysiological techniques in the context of envenoming treatment and ascertain the extent to which their results can be extrapolated. Despite the inherent challenges, electrophysiology in general, and patch-clamp methods in particular, remain powerful assets in venom and antivenom research, where we predict that they will continue to provide valuable molecular insights that can be exploited in the development of effective treatments for ion channel-related pathologies.
